# Bioactivity-guided isolation of rosmarinic acid as the principle bioactive compound from the butanol extract of *Isodon rugosus* against the pea aphid, *Acyrthosiphon pisum*

**DOI:** 10.1371/journal.pone.0215048

**Published:** 2019-06-24

**Authors:** Saira Khan, Clauvis Nji Tizi Taning, Elias Bonneure, Sven Mangelinckx, Guy Smagghe, Raza Ahmad, Nighat Fatima, Muhammad Asif, Mohammad Maroof Shah

**Affiliations:** 1 Department of Biotechnology, COMSATS University Islamabad, Abbottabad Campus, Abbottabad, Pakistan; 2 Department of Plants and Crops, Faculty of Bioscience Engineering, Ghent University, Ghent, Belgium; 3 Department of Green Chemistry and Technology, Faculty of Bioscience Engineering, Ghent University, Ghent, Belgium; 4 Department of Pharmacy, COMSATS University Islamabad, Abbottabad Campus, Abbottabad, Pakistan; 5 Department of Management Sciences, COMSATS University Islamabad, Abbottabad Campus, Abbottabad, Pakistan; Al-Azhar University, EGYPT

## Abstract

Aphids are agricultural pest insects that transmit viruses and cause feeding damage on a global scale. Current pest control practices involving the excessive use of synthetic insecticides over many years have resulted in aphid resistance to a number of pesticides. In nature, plants produce secondary metabolites during their interaction with insects and these metabolites can act as toxicants, antifeedants, anti-oviposition agents and deterrents towards the insects. In a previous study, we demonstrated that the butanol fraction from a crude methanolic extract of an important plant species, *Isodon rugosus* showed strong insecticidal activity against the pea aphid, *Acyrthosiphon pisum*. To further explore this finding, the current study aimed to exploit a bioactivity-guided strategy to isolate and identify the active compound in the butanol fraction of *I*. *rugosus*. As such, reversed-phase flash chromatography, acidic extraction and different spectroscopic techniques were used to isolate and identify the new compound, rosmarinic acid, as the bioactive compound in *I*. *rugosus*. Insecticidal potential of rosmarinic acid against *A*. *pisum* was evaluated using standard protocols and the data obtained was analyzed using qualitative and quantitative statistical approaches. Considering that a very low concentration of this compound (LC_90_ = 5.4 ppm) causes significant mortality in *A*. *pisum* within 24 h, rosmarinic acid could be exploited as a potent insecticide against this important pest insect. Furthermore, *I*. *rugosus* is already used for medicinal purposes and rosmarinic acid is known to reduce genotoxic effects induced by chemicals, hence it is expected to be safer compared to the current conventional pesticides. While this study highlights the potential of *I*. *rugosus* as a possible biopesticide source against *A*. *pisum*, it also provides the basis for further exploration and development of formulations for effective field application.

## Introduction

Aphids are among the most important agricultural pest insects of many crops worldwide. They feed exclusively on plant phloem sap by inserting their needle-shaped mouthparts into sieve elements, usually resulting to plant discoloration, stunting and deformation. Honey dew produced by aphids promotes the growth of sooty molds which further reduces the economic value of the crop [1, 2]. Moreover, aphids are also vectors of many important plant viruses [3–5]. The pea aphid, *Acyrthosiphon pisum* (Hemiptera: Aphididae), adversely affects economically important legume crops worldwide. It is oligophagous, comprising of a number of biotypes or races living on a number of legume hosts (red clover, pea, broad bean and alfalfa races) [6–9]. Current aphid control strategies predominantly rely on the use of insecticides such as carbamates, organophosphates, pyrethroids, neonicotinoids and pymetrozine [10]. However, the repeated use of these insecticides for many years has resulted in aphid resistance to most insecticides, making it very difficult to control aphids [11].

The use of botanical pesticides could present a safe alternative compared to the use of broad spectrum chemical insecticides in crop protection [12, 13]. In nature, plants produce secondary metabolites during their interaction with insects and these metabolites can act as toxicants [14], antifeedants [15], anti-oviposition agents and deterrents towards insects [16]. Because of such wide insecticidal properties, the study of secondary metabolites and the development of new potent formulations based on them have become increasingly important. For the discovery of bioactive natural products against insect pests, the screening of plant extracts followed by bioactivity-guided fractionation, isolation and identification of active principles is considered to be one of the most successful strategies [17].

*Isodon rugosus* (Wall. ex Benth.) Codd (syn. *Plectranthus rugosus* Wall. ex. Benth.) is an aromatic branched shrub, belonging to the Lamiaceae family. The plant is used in Pakistani traditional medicine for many diseases as an antiseptic, hypoglycemic, antidiarrheal and as bronchodilator [18, 19]. Among many other traditional medicinal uses, the plant extracts and different solvent fractions are known to be effective as antifungal [20], antibacterial, phytotoxic [21] and antioxidant agents [22] and are able to show lipoxygenase inhibitory activities [23]. Based on phytochemical studies, this plant is known to contain steroids, terpenoids, saponins, flavonoids, tannins, coumarins, cardiac glycosides, β-cyanin and reducing sugars [24]. Diterpenoids (effusanin-A, rugosinin, effusanin-B, oridonin, effusanin-E and lasiokaurin) [25] and triterpenoids (acetyl plectranthoic acid, plectranthoic acid A and B and plectranthadiol) have also been successfully isolated from this plant [26]. However, despite several studies on the bioactivity of *I*. *rugosus*, where most efforts were focused towards human health, none of these have isolated and analyzed the insecticidal activity of compounds from this plant.

In a previous study, we evaluated the aphicidal properties of the hexane, dichloromethane, butanol and ethyl acetate fractions of a crude methanolic extract from *I*. *rugosus*, and confirmed that the butanol fraction showed the best activity against the pea aphid, *A*. *pisum* [27]. To further explore this finding, a bioactivity-guided strategy against *A*. *pisum* was used to isolate and identify the active compound in the butanol fraction of *I*. *rugosus*.

## Materials and methods

### Insects

A continuous colony of *A*. *pisum* was maintained on faba bean plants (*Vicia faba*) in the Laboratory of Agrozoology at Ghent University, Belgium at 23–25 °C and 65±5% relative humidity (RH) under a 16:8 h light: dark photoperiod [28]. All the bioassays were performed under these conditions. Neonates (< 24 h old) of *A*. *pisum* were used for all the bioassays. Mortality was observed after 24 h of treatment by slight probing of the aphids with the help of a brush and also by analyzing post-mortem color change of the body.

### Plant collection and extraction

The aerial parts of *I*. *rugosus* were collected from lower Northern areas of Pakistan in the month of October, 2012. The plant material was shade-dried for up to 3 months and ground to powder using an electric grinder. Extracts were prepared as described by Khan et al. [27, 29]. Briefly, 1 kg of the dried powder was soaked in a glass jar containing 3 L of methanol at room temperature. After two days, the solvent layer was filtered with a Whatman filter paper No. 1 and this procedure was repeated three times. By using a rotary evaporator, the obtained filtrate was concentrated at 35 °C and the resulting crude methanolic extract was stored at 4 °C. For fractionation, 90 g dried crude methanolic extract was mixed with five parts of water and then extracted successively by n-hexane (4 × 150 mL), dichloromethane (4 × 150 mL), ethyl acetate (4 × 150 mL) and n-butanol (4 × 150 mL) as described by Khan et al. [27]. All the fractions were concentrated using a rotary evaporator under reduced pressure at 40 °C. The resulting extracts were stored in a refrigerator at 4 °C until further use.

### Isolation of the bioactive principle

Based on bioassays conducted by Khan et al. [27], the butanol extract presented the best biological activity against *A*. *pisum* and was hence selected in this study for further bioactivity-guided fractionation and identification of the active principle. The butanol extract (500 mg) was eluted with a Reveleris automated flash chromatography instrument on a 12 g C18 pre-packed column (GRACE, Columbia, MD, US) starting with 100% water. The gradient was ramped to 100% methanol over 60 column volumes (CV) and after collection of 95 fractions, the solid phase was flushed with 5 CV acetonitrile. The flow rate was set to 30 mL/min (S1 Table). Based on the UV spectral data, the 95 fractions were combined into a total of 14 subfractions. These combined fractions were evaporated under reduced pressure at 45 °C and finally under high vacuum, resulting in 14 subfractions (1A- 14A, S2 Table). The 14 subfractions were evaluated for their bioactivity against *A*. *pisum*, of which, one active fraction was selected on the basis of maximum bioactivity for further fractionation through preparative liquid chromatography (prep-LC). A 10% solution of this active fraction was prepared in methanol. Two solvents were used, water (solvent A) and acetonitrile (solvent B). A gradient was set starting with 100% solvent A from 0 to 100 min. From 100 min to 110 min, solvent B went from 18% to 100% and stayed at 100% until 128 min, and then to 0% at 128.10 min and stayed at 0% until 132.10 min. After concentration under reduced pressure with a rotary evaporator and finally under high vacuum, three fractions, 3A-1, 3A-2 and 3A-3 were obtained. Fraction 3A-3 was selected for active compound identification (NMR and LC-MS) on the basis of the bioactivity against *A*. *pisum*. This compound was obtained in pure form by doing a second flash chromatographic separation of 5 g of butanol extract and by using the run conditions as mentioned in S3 Table. From the second flash chromatography, a total of 354 fractions were collected which were combined into six fractions, 1B, 2B, 3B, 4B, 5B and 6B on the basis of UV spectra and were further analyzed for their bioactivity after concentration with a rotary evaporator under reduced pressure and high vacuum (S4 Table). The most active fraction was selected for further purification on the basis of best bioactivity. On the basis of knowledge regarding the acidic compound present in sub fraction 3A-3 (from ^1^H NMR and HPLC-MS analysis), an extraction under acidic conditions was done to isolate the active compound from the active fraction from the second flash chromatography. For this purpose, 200 mg of this fraction was dissolved in 10 mL of distilled water and acidified with 4 drops of hydrochloric acid (12 M). Following extraction with ethyl acetate (four times 5 mL), two phases, ethyl acetate and aqueous, were obtained. Both the ethyl acetate and the aqueous phase were concentrated and analyzed for their bioactivity. Last traces of ethyl acetate were removed azeotropically with toluene and evaporation under high vacuum of the residues resulted in 60 mg from the ethyl acetate phase and 60 mg from the aqueous phase. The purified active principle was identified through different spectroscopic techniques.

### Identification of the bioactive compound

Mass spectra were recorded using a HPLC-MS instrument consisting of an Agilent (Waldbronn, Germany) model 1100 liquid chromatograph with a diode array detector coupled with a mass spectrometer with electrospray ionization geometry (Agilent MSD 1100 series). The prep-LC consisted of an Agilent 1100 Series liquid chromatograph using a Supelco Ascentis C18 column (I.D. x L 21.2 mm x 150 mm, 5 μm particle size) connected to an UV-VIS variable wavelength detector (VWD) and automatic fraction collector. Flash chromatography was performed with the Reveleris Flash System (GRACE). ^1^H and ^13^C NMR spectra were obtained on a BRUKER Advance III 400 spectrometer. All the solvents and chemicals used were of analytical grade. Optical rotation was taken with a JASCO P-2000 series polarimeter.

### Insecticidal bioactivity

For the bioassays, artificial diet test cages were constructed according to Sadeghi et al. [30]. Between two layers of parafilm, 100 μL of liquid artificial diet was sealed. On these layers of parafilm, ten neonate aphids were placed and to avoid the escape of aphids, the cages were covered with a hollow plastic ring incorporating a ventilated lid. In six aerated well plates, these cages were kept in an inverted position. Five concentrations were used for each treatment against the aphids. A stock solution of 1% was prepared by mixing 1 mg of each fraction in 100 μL of water. For reversed-phase flash fractions, five concentrations of 50, 25, 12.5, 6.3 and 3.1 ppm and for prep-LC and acidic extraction fractions, five concentrations of 5, 2.5, 1.3, 0.7 and 0.3 ppm, were prepared by diluting the stock solution with the artificial diet of aphids. For each concentration, a final volume of 300 μL was made to carry out three replications of each treatment (100 μL for each replication). Pure isolated and identified active compound was analyzed in eight different concentrations, including 50, 25, 12.5, 6.3, 3.1, 1.6, 0.8 and 0.4 ppm, by using a stock solution of 1 mg of the compound in 100 μL of water. The untreated artificial diet was used as a control and for each treatment three replications were used in all the bioassays. Mortality was analyzed after 24 h of each treatment.

Additionally, the growth of the surviving aphids exposed to 0.4 ppm of the active compound for 24 h was followed for 9 days (on the same treated diet) in comparison to the untreated aphids.

### Data analysis

For statistical analysis, Probit analysis of mortality vs. concentration using POLO-Plus program version 2 was conducted and the lethal concentrations (LC_50_, LC_90_) and their corresponding 95% confidence intervals (95% CI) were estimated for each fraction. LC’s were considered to be significantly different when the 95% CI’s did not overlap.

## Results

### Bioactivity of fractions from the butanol extract of *I*. *rugosus*

Bioactivity of the fourteen fractions (1A-14A) obtained through the first reversed-phase flash chromatography of 500 mg of butanol extract of *I*. *rugosus* was analyzed for 24 h against *A*. *pisum*. Except fractions 8A, 9A, 11A, 13A and 14A, all the other fractions showed considerable toxic effects against *A*. *pisum*. Among all the fractions, fraction 3A was the most active fraction with lower LC’s values (Table 1).

**Table 1:** Toxicity of subfractions of the butanol fraction from first reversed-phase flash chromatography against newborn (< 24 h old) *Acyrthosiphon pisum* nymphs following 24 h exposure to artificial diet containing different concentrations of the subfractions.

Fractions	LC_50_ (95% CI) ppm	Ratio	LC_90_ (95% CI) ppm	Ratio	Slope ± SE	Chi-Square	HF
1A	5.5 (3–8) a	2.6	66 (37–211) a	2.2	1.1 ± 0.3	7.1	0.5
2A	8.9 (6.1–12) a	4.2	81 (47–231) a	2.7	1.3 ± 0.3	5.6	0.4
3A	2.1 (0.6–3.8) a	1.0	30 (18–85) a	1.0	1.1± 0.3	7.5	0.6
4A	6.8 (3.8–10) a	3.2	112.2 (54–561) a	3.8	1.1 ± 0.3	4.6	0.4
5A	3.3 (1.3–5.4) a	1.6	50 (28–176) a	1.7	1.1 ± 0.3	10.1	0.8
6A	18 (13–27) b	8.5	187 (90–808) a	6.3	1.3 ± 0.3	3.8	0.3
7A	74 (52–169) c	35.3	267 (131–1651) a	9.1	2.3 ± 0.6	8.1	0.6
8A	-	-	-	-	1.7 ± 0.7	7.0	0.5
9A	-	-	-	-	2.0 ± 1.3	4.7	0.4
10A	36 (33–40) d	17.2	52.5 (46–64) a	1.8	8.0 ± 1.4	2.8	0.2
11A	-	-	-	-	1.6 ± 0.6	8.5	0.7
12A	51 (43–71) c	24.5	109 (77–241) a	3.7	3.9 ± 1.0	2.2	0.2
13A	-	-	-	-	1.5 ± 1.2	6.6	0.5
14A	-	-	-	-	2 ± 1.3	4.7	0.4

Data is presented as lethal concentration values, 50% (LC50) and 90% (LC90) (both in ppm) together with their particular 95% confidence interval (95% CI), the slope ± SE of the toxicity vs concentration curve, and the Chi-Square and heterogeneity factor HF as accuracy of data fitting to probit analysis in POLO-PlusV2. Due to non-overlapping of 95% CI, different letters in the same column indicate significant differences. Ratio, LCx, fraction/LCx, 3A

### Bioactivity of subfractions from fraction 3A collected through prep-LC

The three collected subfractions (3A-1, 3A-2 and 3A-3) of 3A were analyzed against *A*. *pisum* for 24 h. Fraction 3A-1 and fraction 3A-2 gave negligible toxic effects (no LC_50_ and LC_90_). Fraction 3A-3 was the most toxic fraction analyzed against *A*. *pisum* with low LC’s values (Table 2).

**Table 2:** Toxicity of subfractions of fraction 3A against newborn (< 24 h old) *Acyrthosiphon pisum* nymphs following 24 h exposure to artificial diet containing different concentrations of the subfractions.

Fractions	LC_50_ (95% CI) ppm	Ratio	LC_90_ (95% CI) ppm	Ratio	Slope ± SE	Chi-Square	HF
3A-1	-	-	-	-	2.0 ± 1.3	4.9	0.4
3A-2	-	-	-	-	1.5 ± 1.2	6.6	0.5
3A-3	1 (0.6–1.6)	1	14 (6.1–97)	1	1.1± 0.3	14.8	1.1

Data is presented as lethal concentration values, 50% (LC50) and 90% (LC90) (both in ppm) together with their particular 95% confidence interval (95% CI), the slope ± SE of the toxicity vs concentration curve, and the Chi-Square and heterogeneity factor HF as accuracy of data fitting to probit analysis in POLO-PlusV2. Ratio, LCx, fraction/LCx, 3A-3

#### Spectroscopic analysis of fraction 3A-3

Out of three subfractions of 3A (3A-1, 3A-2 and 3A-3), fraction 3A-3 was the most bioactive fraction against *A*. *pisum*. This fraction 3A-3 was analyzed through ^1^H NMR which confirmed that it contained rosmarinic acid. Different gradients were used to purify the compound but during different Prep-LC runs, the chromatographic behavior, that is, peak shape and position, of this fraction was inconsistent. Therefore, the reversed-phase flash chromatography was repeated with 5 g of butanol fraction of *I*. *rugosus* in order to get the most bioactive compound in pure form.

### Bioactivity of fractions of butanol extract from the second reversed-phase flash chromatography

Six fractions (1B-6B) obtained through a second reversed-phase flash chromatography of the butanol extract of *I*. *rugosus*, were analyzed against *A*. *pisum* for 24 h. Out of the six fractions analyzed, fraction 4B, 5B and 6B showed negligible toxicity (no LC_50_ and LC_90_). Fraction 1B was more toxic and moderate toxicity was observed for fraction 2B. Lower toxicity was found for fraction 3B (Table 3).

**Table 3:** Toxicity of subfractions of the butanol fraction from a second reversed-phase flash chromatography against newborn (<24 h old) *Acyrthosiphon pisum* nymphs following 24 h exposure to artificial diet containing different concentrations of the subfractions.

Fractions	LC_50_ (95% CI) ppm	Ratio	LC_90_ (95% CI) ppm	Ratio	Slope ± SE	Chi-Square	HF
1B	2.5 (1–4.1) a	1.0	28 (18–69) a	1	1.2 ± 0.3	11.4	0.9
2B	7.5 (4.3–11) b	3.0	71 (38–280) a	2.5	1.3 ± 0.3	16.5	1.3
3B	16 (11–26) c	6.5	101 (52–417) a	3.6	1.6± 0.3	22.3	1.7
4B	-	-	-	-	1.0 ± 0.3	25.3	2.0
5B	-	-	-	-	1.5 ± 1.2	6.6	0.5
6B	-	-	-	-	1.8 ± 0.7	6.5	0.5

Data is presented as lethal concentration values, 50% (LC50) and 90% (LC90) (both in ppm) together with their particular 95% confidence interval (95% CI), the slope ± SE of the toxicity vs concentration curve, and the Chi-Square and heterogeneity factor HF as accuracy of data fitting to probit analysis in POLO-PlusV2. Due to non-overlapping of 95% CI, different letters in the same column indicate significant differences. Ratio, LCx, fraction/LCx, 1B

### Bioactivity of the ethyl acetate and aqueous phase of acidic extraction

Both collected phases of acidic extraction were analyzed for their insecticidal potential through bioassays against *A*. *pisum* for 24 h. The aqueous phase caused negligible toxic effects (no LC_50_ and LC_90_) while the ethyl acetate phase caused more toxicity (Table 4).

**Table 4:** Toxicity of ethyl acetate and aqueous phase of acidic extraction against newborn (< 24 h old) *Acyrthosiphon pisum* nymphs following 24 h exposure to artificial diet containing different concentrations of both phases.

Fractions	LC_50_ (95% CI) ppm	Ratio	LC_90_ (95% CI) ppm	Ratio	Slope ± SE	Chi-Square	HF
Aqueous	-	-	-	-	1.5 ± 1.2	6.6	0.5
Ethyl acetate	0.2 (0.04–0.5)	1	9.2 (3.9–13)	1	0.8 ± 0.3	4.2	0.3

Data is presented as lethal concentration values, 50% (LC50) and 90% (LC90) (both in ppm) together with their particular 95% confidence interval (95% CI), the slope ± SE of the toxicity vs concentration curve, and the Chi-Square and heterogeneity factor HF as accuracy of data fitting to probit analysis in POLO-PlusV2. Ratio, LCx, fraction/LCx, ethyl acetate

### Identification of the most bioactive compound

Out of the two phases of acidic extraction, the ethyl acetate phase fraction was the most active. After removing ethyl acetate azeotropically, this fraction was analyzed and the active compound was identified as rosmarinic acid through HPLC-MS, optical rotation measurement and ^1^H and ^13^C NMR spectroscopy.

#### HPLC-MS

**Fig 1:**
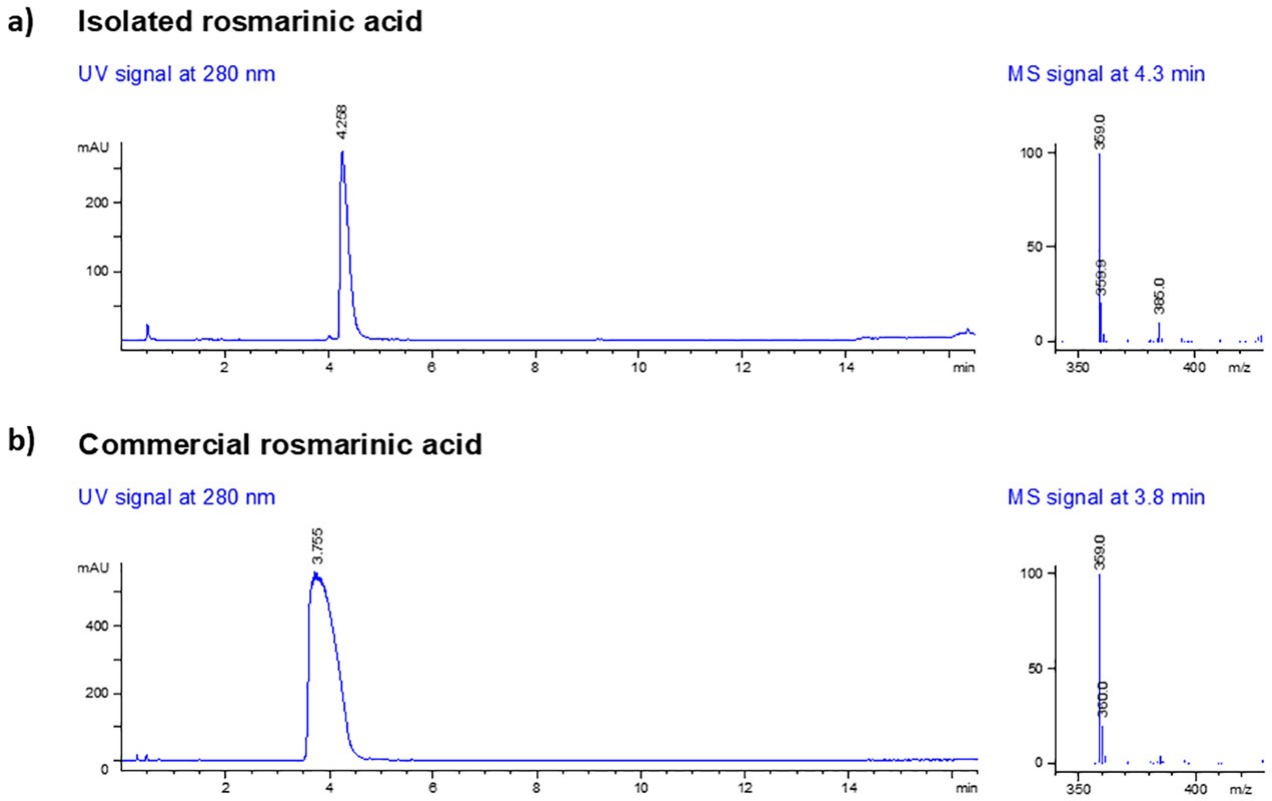
Mass spectra (negative mode electrospray ionization) of rosmarinic acid obtained via HPLC-MS with a pseudo molecular ion at *m/z* value of 359 (a) isolated rosmarinic acid (b) commercial rosmarinic acid.

Both isolated and commercial rosmarinic acid (Sigma Aldrich) had the same peak appearance in the HPLC-MS chromatograms with the same solvent gradient. Both had a pseudo-molecular ion with an *m/z* value of 359 with negative mode electrospray ionization which confirmed that it was rosmarinic acid (Fig 1).

#### Optical rotation and ^1^H and ^13^C NMR

**Fig 2:**
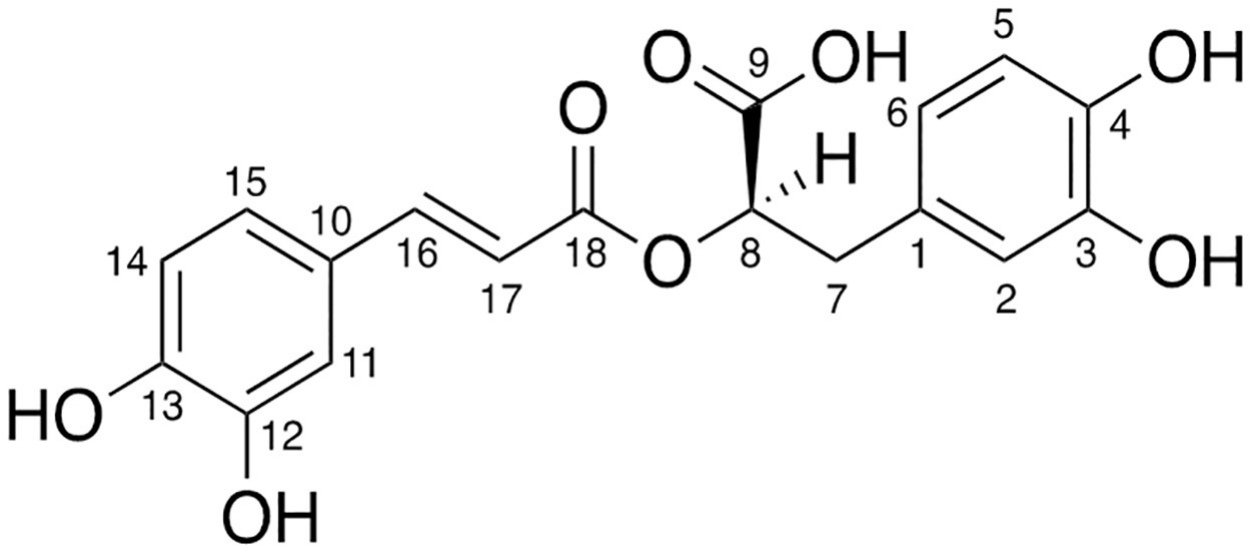
Structure of rosmarinic acid isolated from *I*. *rugosus*.

Brown crystals; ${\left[\alpha \right]}_{D}^{24}+78.0{}^{\circ}$ (*c* 0.233, MeOH); ^**1**^**H NMR (400 MHz, CD**_**3**_**OD): δ°**3.01 (1H, dd, *J* = 8.3, 14.3 Hz, H^7a^), 3.10 (1H, dd, *J* = 4.4, 14.3 Hz, H^7b^), 5.19 (1H, dd, *J* = 4.4, 8.3 Hz, H^8^), 6.27 (1H, d, *J* = 15.9, H^17^), 6.61 (1H, dd, *J* = 2.0, 8.0 Hz, H^6^), 6.70 (1H, d, *J* = 8.0 Hz, H^5^), 6.75 (1H, d, *J* = 2.0 Hz, H^2^), 6.78 (1H, d, *J* = 8.2 Hz, H^14^), 6.95 (1H, dd, *J* = 2.0, 8.2 Hz, H^15^), 7.04 (1H, d, *J* = 2.0 Hz, H^11^), 7.55 (1H, d, *J* = 15.9 Hz, H^16^);^**13**^**C NMR (100 MHz, CD**_**3**_**OD): δ**°37.9 (C^7^), 74.6 (C^8^), 114.4 (C^17^), 115.2 (C^11^), 116.3 (C^5^), 116.5 (C^14^), 117.6 (C^2^), 121.8 (C^6^), 123.2 (C^15^), 127.7 (C^10^), 129.2 (C^1^), 145.3 (C^4^), 146.2 (C^3^), 146.8 (C^12^), 147.7 (C^16^), 149.7 (C^13^), 168.4 (C^18^), 173.5 (C^9^); **ESI-MS**: *m/z* (%) 359 (M-H^+^, 100). Optical rotation and NMR data were in accordance with the literature (Fig 2) [31, 32].

### Bioactivity of *I*. *rugosus* rosmarinic acid and commercial rosmarinic acid

Rosmarinic acid isolated from *I*. *rugosus* and commercial rosmarinic acid (Sigma Aldrich) were analyzed against *A*. *pisum* for their pesticidal activity for 24 h. Both *I*. *rugosus* rosmarinic acid (RA) and commercial RA caused comparable toxic effects in the treated aphids (Table 5).

**Table 5:** Toxicity of isolated rosmarinic acid (RA) and commercial rosmarinic acid (RA) against newborn (< 24 h old) *Acyrthosiphon pisum* nymphs following 24 h exposure to artificial diet containing different concentrations of isolated rosmarinic acid and commercial rosmarinic acid.

Compound	LC_50_ (95% CI) ppm	Ratio	LC_90_ (95% CI) ppm	Ratio	Slope ± SE	Chi-Square	HF
Commercial RA	0.2 (0.05–0.5) a	1	14 (7.4–42) a	2.6	0.7 ± 0.2	15.5	0.7
*I*. *rugosus* RA	0.2 (0.04–0.4) a	1	5.4 (3.3–12) a	1	0.8 ± 0.2	10.5	0.5

Data is presented as lethal concentration values, 50% (LC50) and 90% (LC90) (both in ppm) together with their particular 95% confidence interval (95% CI), the slope ± SE of the toxicity vs concentration curve, and the Chi-Square and heterogeneity factor HF as accuracy of data fitting to probit analysis in POLO-PlusV2. Due to overlapping of 95% CI, same letter in the same column indicate no significant differences. Ratio, LCx, compound/LCx, *Isodon rugosus* RA

### Comparison of the growth of surviving aphids exposed to rosmarinic acid-treated and untreated diet after 24 h of bioassay

**Fig 3:**
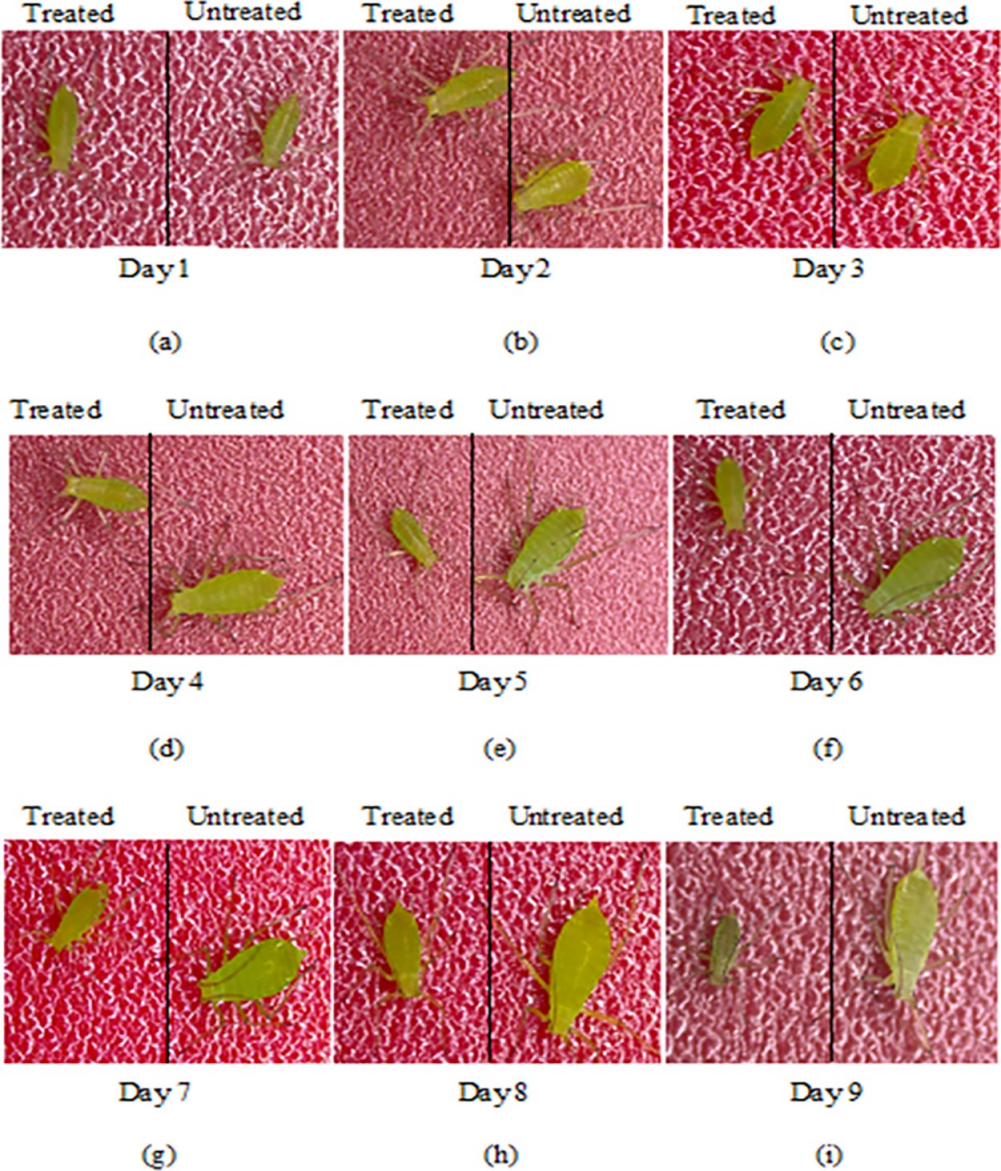
Comparison between growth of surviving aphids exposed to rosmarinic acid-treated and untreated diet after 24 h of bioassay, (a) to (i) comparison observed for up to 9 days, all treated aphids died by day 9.

After incorporating rosmarinic acid into the aphid’s diet at a concentration of 0.4 ppm, its effect on *A*. *pisum* that survived after 24 h treatment, was analyzed every day for up to 9 days (on same treated diet). It was confirmed that rosmarinic acid had a drastic effect on their growth. Firstly, most aphids exposed to treated diet were dead while the survivors did not grow further to become adults and were thus not able to reproduce further. Fig 3 shows a comparison between treated and untreated aphids. There was a clear difference between untreated and treated aphids after day 4, and by day 9 the treated aphids were all dead, while the untreated aphids were still alive.

## Discussion

Screening candidate plants, purifying active ingredients, isolating and identifying the active plant constituents is required to discover new bioactive natural products [33]. We applied this methodology to identify rosmarinic acid as an active principle from the plant *I*. *rugosus*. Based on our previous study on the insecticidal activity of botanical extracts from various plant species, we found that the extract from *I*. *rugosus* was the most toxic to *A*. *pisum* [27] Further fractionation showed that the butanol fraction most likely contained the active principle. In this study we used the bioactivity-guided strategy to isolate and identify the active compound as rosmarinic acid. This strategy is interesting and has been used in previous studies to identify bioactive compounds. For example, the butanol fraction from *Citrullus colocynthis* was reported to be active against the black legume aphid, *Aphis craccivora*, and through the bioactivity-guided isolation strategy, the active principle, 2-O-ß-D-glucopyranosylcucurbitacin E, was successfully isolated [34]. Similarly, in another study involving bioactivity-guided isolation, the active principle, ailanthone, was isolated from the aqueous fraction of *Ailanthus altissima* against *A*. *pisum* [35].

In this study, the butanol fraction was subfractionated through reversed-phase flash chromatography. After bioactivity based screening of all the resulting subfractions (1A-14A) against *A*. *pisum*, fraction 3A with lower LC values was selected for further fractionation. Through prep-LC, fraction 3A was subfractionated and the resulting subfractions (3A-1, 3A-2 and 3A-3) were analyzed for their bioactivity. Fraction 3A-3 with lower LC values was subjected to spectroscopic analysis. ^1^H NMR spectroscopy confirmed that the isolated fraction contained rosmarinic acid. However, due to the inconsistent chromatographic behavior during prep-LC, not enough compound could be collected to record ^13^C NMR data. The inconsistent chromatographic behavior with peak splitting observed could have arisen from several causes; a contamination on guard or analytical column inlet, a blocked frit or a small void at the column inlet (~wear). The problem of peak shifting (variable retention times) could have been due to small changes in mobile composition, temperature fluctuations, column overloading or a combination of these problems which could have led to different UV patterns for each run. Due to this problem, the reversed-phase flash chromatography was repeated with a larger amount of the butanol fraction. Out of all the resulting subfractions (1B-6B), 1B was selected with lower LC values against *A*. *pisum*. Fraction 1B was subjected to acidic extraction to get two phases, aqueous and ethyl acetate. The ethyl acetate phase fraction was more active with lower LC values. After removing ethyl acetate, the active principle was identified through different spectroscopic techniques as rosmarinic acid. Similarly in another study, Chakraborty et al. [36] reported the isolation of caffeic acid and rosmarinic acid from *Basilicum polystachyon* through acidic extraction with HCl followed by partitioning with ethyl acetate and analyzed their antimicrobial activities.

This study reports the isolation and purification of rosmarinic acid (RA) from *I*. *rugosus* and its bioactivity against *A*. *pisum* for the first time. Feeding bioassays were used to analyze the toxicity of rosmarinic acid. For evaluating the insecticidal activity, incorporation of these products into a food source is a standard technique. Under controlled conditions, the use of artificial diet permits testing of a small amount of insecticidal product and stimulates aphids to oral exposure easily. This technique is fast, easy to handle, inexpensive and gives results in a short period of time including effects of insecticides on aphids [30]. In the current study, it was observed that the aphids were feeding on the treated diet, which is why it was concluded that aphids were dying because of toxic effects of rosmarinic acid.

In this study, there was no significant difference observed between the bioactivity depicted by both isolated and commercial rosmarinic acid. *Isodon rugosus* rosmarinic acid gave LC values of LC_50_ = 0.2 ppm and LC_90_ = 5.4 ppm. These are very low LC values depicted after 24 h of bioassay and such low LC values have not been previously reported in any study with compounds against *A*. *pisum* using the same feeding bioassay methodology [30, 37–39, 28, 40, 41]. This means that a very low amount of rosmarinic acid can cause significant toxic effects against *A*. *pisum* in 24 h. Furthermore, many plant essential oils have caused over 90% mortality in *A*. *pisum* upon application of 2 μl l^-1^ of oil in air using fumigation assays, with most of the plant species belonging to the Lamiacea family [42]. *I*. *rugosus* also belongs to the Lamiacea family, indicating that plants from this family could have a high potential to control the aphid, *A*. *pisum* through integral valorization of different plant constituents. Based on this observation, the essential oil from *I*. *rugosus* should be studied as well for possible control of these aphids.

Very few insecticidal activities have been reported for rosmarinic acid. Regnault-Roger et al, [43] investigated the insecticidal activities of polyphenolic compounds, isolated from five plants belonging to Lamiaceae family against *Acanthoscelides obtectus* (Say) and observed that among all the polyphenolic compounds, rosmarinic acid and luteolin-7-glucoside were more toxic. However, in another study, rosmarinic acid exhibited negligible toxicity against the red palm weevil larvae, *Rhynchophorus ferrugineus* [44]. Although the mode of action of rosmarinic leading to toxicity in insects is unknown, one could postulate that the low toxicity observed in the red palm weevil could have arisen from several possible reasons probably linked to its genotype and gene expression profile as observed in other insects tolerant to some classical insecticides.

Additionally, a comparison between the growth of surviving aphids exposed to rosmarinic acid-treated and untreated diet after 24 h of bioassay was analyzed. It was clearly observed that the growth of surviving *A*. *pisum* nymphs stopped after 48 h of exposure to rosmarinic acid-treated diet, resulting in a size reduction and ultimately death as compared to aphids exposed to an untreated diet. A similar observation was made by Sadeghi et al. [30] who observed that the aphid size was reduced after 48 h of exposure to novel biorational insecticides, flonicamid and pymetrozine, and mortality was observed after 72 h.

## Conclusion

In this study, *I*. *rugosus* was identified as an interesting source for a botanical insecticide against *A*. *pisum*. Following bioactivity-guided selection, rosmarinic acid was isolated and identified through spectroscopic analysis as the bioactive compound in the *I*. *rugosus* extract for the first time. Based on the bioassay results, either the extracts from *I*. *rugosus* or the isolated insecticidal compound, rosmarinic acid could be used to develop effective aphicides, because of the high mortality of aphids caused at very low rosmarinic acid concentrations in 24 h. This potential botanical insecticide may fit well in integrated pest management programs intended to control aphids. Considering that *I*. *rugosus* is known to be used for medicinal purposes, it is expected to be safer as compared to the current conventional pesticides used for controlling aphids. Also, rosmarinic acid is known to reduce genotoxic effects induced by chemicals, which is contrary to some currently used toxic synthetic pesticides that could induce genotoxic effects in consumers. An interesting avenue to follow for future studies will be the analyses of the underlying molecular mechanisms responsible for the cause of mortality in rosmarinic acid-treated aphids. While this study highlights the potential of *I*. *rugosus* as a possible biopesticide source against a notorious insect pest such as *A*. *pisum*, it also provides the basis for further exploration and development of a formulation for effective field application. However, more experiments under field conditions are required to further verify the applicability of rosmarinic acid for the insect’s control and in future contact application tests can be performed to broaden its effects on crops under field conditions. From an implementation point of view, genes involved in the biosynthesis of rosmarinic acid could be transformed under the control of a phloem-specific promoter to produce resistant crops towards aphids.

## Supporting information

S1 Table First reversed-phase flash chromatography conditions of butanol fraction (500 mg) from *Isodon rugosus*. (DOCX)

S2 Table Subfractions (1A-14A) collected from the first reversed-phase flash chromatography of butanol extract (500 mg). (DOCX)

S3 Table Second reversed-phase flash chromatography conditions of butanol fraction (5 g) of *Isodon rugosus*. (DOCX)

S4 Table Subfractions (1B-6B) from the second reversed-phase flash chromatography of butanol extract (5 g). (DOCX)

## References

[1] Kim SK, Kim YC, Lee S, Kim JC, Yun MY, Kim IS. Insecticidal activity of rhamnolipid isolated from Pseudomonas. EP-3 against green peach aphid (*Myzus persicae*). J Agric Food Chem. 2011; 59: 934–938 10.1021/jf104027x 21192722

[2] Baek MY, Park HJ, Kim GM, Lee DY, Lee GY, Moon SJ et al Insecticidal alkaloids from the seeds of Macleaya cordata on cotton aphid (*Aphis gossypii*). J Korean Soc Appl Biol Chem. 2013; 56: 135–140.

[3] Sylvester ES. In Aphids, Their Biology, Natural Enemies and Control (2C): Viruses Transmitted by Aphids; Minks A. K., Harrewijn P., Eds.; Elsevier: Amsterdam, The Netherlands 1989; 65–87.

[4] James CKNG, Perry KL. Transmission of plant viruses by aphid vectors. Mol Plant Pathol. 2004; 5: 505–511. 10.1111/j.1364-3703.2004.00240.x 20565624

[5] Dedryver CA, Le Ralec A, Fabre F. The conflicting relationships between aphids and men: A review of aphid damage and control strategies. C R Biol. 2010; 333(6–7): 539–553. 10.1016/j.crvi.2010.03.009 20541165

[6] Via S, Shaw AJ. Clonal genetic variability and short term evolution in the size and shape of pea aphids. Evolution. 1996; 50: 163–173. 28568861 10.1111/j.1558-5646.1996.tb04483.x

[7] Via S. Reproductive isolation between sympatric races of pea aphids. I Gene flow restriction and habitat choice. Evolution. 1999; 53:1446–1457. 10.1111/j.1558-5646.1999.tb05409.x 28565574

[8] Peccoud J, Simon JCH, McLaughlin HJ, Moran NA. Postpleistocene radiation of the pea aphid complex revealed by rapidly evolving endosymbionts. Proc Natl Acad Sci USA. 2009a; 106: 16315–16320. 19805299 10.1073/pnas.0905129106 PMC2752580

[9] Peccoud J, Ollivier A, Plantegenest M, Simon JCHA. Continuum of genetic divergence from sympatric host races to species in the pea aphid complex. Proc Natl Acad Sci USA. 2009b; 106: 7495–7500. 19380742 10.1073/pnas.0811117106 PMC2678636

[10] Sparks TC, Nauen R. IRAC: mode of action classification and insecticide resistance management. Pestic Biochem Physiol. 2015; 121: 122–128. 10.1016/j.pestbp.2014.11.014 26047120

[11] Jaouannet M, Rodriguez PA, Thorpe P, Lenoir CJ, MacLeod R, Escudero-Martinez C, Bos JI. Plant immunity in plant–aphid interactions. Front Plant Sci. 2014; 5: p.663 10.3389/fpls.2014.00663 25520727 PMC4249712

[12] Isman MB. The role of botanical insecticides, deterrents, and repellents in modern agriculture and an increasingly regulated world. Annu Rev Entomol. 2006; 51: 45–66. 10.1146/annurev.ento.51.110104.151146 16332203

[13] Akhtar Y, Yu Y, Isman MB, Plettner E. Dialkoxybenzene and dialkoxyallylbenzene feeding and oviposition deterrents against the cabbage looper, Trichoplusiani: potential insect behavior control agents. J Agric Food Chem. 2010; 58: 4983–4991. 10.1021/jf9045123 20225858

[14] Pavela R. Larvicidal effects of various Euro-Asiatic plants against *Culex quinquefasciatus* Say larvae (Diptera: Culicidae). Parasitol Res, 2008; 102(3): p.555 10.1007/s00436-007-0821-3 18058128

[15] Akhtar Y; Isman MB. Comparative growth inhibitory and antifeedant effects of plant extracts and pure allelochemicals on four phytophagous insect species. J Appl Entomol. 2004; 128(1): pp.32–38.

[16] Basukriadi A. Wilkins RM. Oviposition deterrent activities of *Pachyrhizus erosus* seed extract and other natural products on *Plutella xylostella* (Lepidoptera: Plutellidae). J Insect Sci. 2014; 14(1): p.244. 25525107 10.1093/jisesa/ieu106 PMC5634021

[17] Archer TL, Segarra E, Bynum ED. Greenbug resistance management on sorghum with insecticide mixtures: a biological and economic analysis. J Econ Entomol. 1999; 92: 794–803.

[18] Sher Z, Khan Z, Hussain F. Ethnobotanical studies of some plants of Chagharzai valley, district Buner, Pakistan. Pak J Bot. 2011; 43: 1445–1452.

[19] Ajmal SM, Mohammad S, Zahid K, Bakht Z. Ethnomedicinal and phytoeconomic elaboration of Lilownai valley, district Shangla Pakistan. IRJP3. 2012; 164–169.

[20] Rauf A, Khan A, Rasool S, Shah ZA, Saleem M. In-vitro antifungal activity of three selected Pakistani medicinal plants. MEJMPR. 2012a; 1: 41–43.

[21] Rauf A, Muhammad N, Khan A, Uddin N, Atif M. Antibacterial and phytotoxic profile of selected Pakistani medicinal plants. WASJ. 2012b; 20: 540–544.

[22] Rauf A, Uddin G, Ali M, Muhammad N, Gul S. Phytochemical screening and antioxidant activity of Pakistani medicinal plants. WJMP. 2013; 2: 1–6.

[23] Janbaz KH, Arif J, Saqib F, Imran I, Ashraf M, et al In-vitro and in-vivo validation of ethnopharmacological uses of methanol extract of *Isodon rugosus* Wall. ex Benth. (Lamiaceae). BMC Complement Altern Med. 2014; 14: 71 10.1186/1472-6882-14-71 24559094 PMC3974051

[24] Razdan TK, Kachroo V, Harkar S, Koul GL. Plectrantholic acids A and B—two new triterpenoids from *Plectranthus rugosus*. Tetrahedron. 1982a; 38: 991–992.

[25] Sun HD, Huang SX, Han QB. Diterpenoids from Isodon species and their biological activities. Nat Prod Rep. 2006; 23: 673–698. 10.1039/b604174d 17003905

[26] Razdan TK, Kachroo V, Harkar S, Koul GL, Dhart KL. Plectranthoic acid, acetyl plectranthoic acid and plectranthadiol-three new triterpenoids from *Plectranthus rugosus*. Phytochemistry. 1982b; 21: 409–412.

[27] Khan S, Taning CNT, Bonneure E, Mangelinckx S, Smagghe G, Shah MM. Insecticidal activity of plant-derived extracts against different economically important pest insects. Phytoparasitica. 2017; 45(1): 113–124.

[28] Nachman RJ, Hamshou M, Kaczmarek K, Zabrocki J, Smagghe G. Biostable and PEG polymer-conjugated insect pyrokinin analogs demonstrate antifeedant activity and induce high mortality in the pea aphid *Acyrthosiphon pisum* (Hemiptera: Aphididae). Peptides. 2012; 34: 266–273. 10.1016/j.peptides.2011.11.009 22108713

[29] Khan S, Shah MM, Ahmad R, Haq IU. The insecticidal potential of botanical extracts for management of Peach fruit fly, *Bactrocera zonata* Saunders, 1842 (Diptera: Tephritidae). Türk entomol derg. 2016; 40 (4): 445–453.

[30] Sadeghi A, Van Damme EJM, Michiels K, Kabera A, Smagghe G. Acute and chronic insecticidal activity of a new mannose-binding lectin from *Allium porrum* against *Acyrthosiphon pisum* via an artificial diet. Can. Entomol. 2009, 141: 95–100.

[31] Dapkevicius A, van Beek TA, Lelyveld GP, van Veldhuizen A, de Groot A, Linssen JP, Venskutonis R. Isolation and Structure Elucidation of Radical Scavengers from *Thymus vulgaris* Leaves. J Nat Prod. 2002; 65(6): 892–896. 12088434 10.1021/np010636j

[32] Hyun HBS, Hrestha S, Boo KH, Cho SK. Evaluation of antioxidant potential of ethyl acetate fraction of *Rosmarinus officinalis* L and its major components. J Korean Soc Appl Bio Chem. 2015; 58(5): 715–722.

[33] Chermenskaya TD, Stepanycheva EA, Shchenikova AV, Savelieva EI, Chakaeva AS. Insecticidal effects of *Ungernia severtzovii* bulb extracts against the grain aphid *Schizaphis graminum* (Rondani). Ind Crops Prod. 2012; 36: 122–126.

[34] Torkey HM, Abou-Yousef HM, Abdel Azeiz AZ, Hoda EAF. Insecticidal effect of cucurbitacin E glycoside isolated from *Citrullus colocynthis* against *Aphis craccivora*. Aust J Basic Appl Sci. 2009; 3(4): 4060–4066.

[35] De Feo V, Mancini E, Voto E, Curini M, Digilio MC. Bioassay-oriented isolation of an insecticide from *Ailanthus altissima*. J Plant Interact. 2009; 4(2): 119–123.

[36] Chakraborty D, Mandal SM, Chakraborty J, Bhattacharyaa PK, Bandyopadhyay A, Mitra A, Gupta K. Antimicrobial activity of leaf extract of *Basilicum polystachyon* (L) *Moench*. Indian J Exp Biol. 2007; 45 (8): 744–748. 17877153

[37] Smagghe G, Mahdian K, Zubrzak P, Nachman RJ. Antifeedant activity and high mortality in the pea aphid *Acyrthosiphon pisum* (Hemiptera, Aphidae) induced by biostable insect kinin analogs. Peptides. 2010; 31: 498–505. 10.1016/j.peptides.2009.07.001 19596392

[38] Carrillo L, Martinez M, Álvarez-Alfageme F, Castanera P, Smagghe G, Díaz I, Ortego F. Abarley cysteine–proteinase inhibitor reduces the performance of two aphid species in artificial diets and transgenic Arabidopsis plants. Transg Res. 2011; 20 (2): 305–319. 10.1007/s11248-010-9417-2 20567901

[39] De Geyter E, Smagghe G, Rahbé Y, Geelen D. Triterpene saponins of *Quillaja saponaria* show strong aphicidal and deterrent activity against the pea aphid *Acyrthosiphon pisum*. Pest Manag. Sci. 2012; 68 (2): 164–169. 10.1002/ps.2235 21717567

[40] Giner M, Avilla J, De Zutter N, Ameye M, Balcells M, Smagghe G. Insecticidal and repellent action of allyl esters against *Acyrthosiphon pisum* (Hemiptera: Aphididae) and *Tribolium castaneum* (Coleoptera: Tenebrionidae). Ind Crop Prod. 2013; 47: 63–68.

[41] Zapata N, Van Damme EJM, Vargas M, Devotto L, Smagghe G. Insecticidal activity of a protein extracted from bulbs of *Phycellaaustralis* Ravenna against the aphids *Acyrthosiphon pisum* Harris and *Myzus persicae* Sulzer. Chil J Agric Res. 2016; 76(2): 188–194.

[42] Ikbal C, Pavela R. Essential oils as active ingredients of botanical insecticides against aphids. J Pest Sci. 2019; 92: 971–986.

[43] Regnault-Roger C, Ribodeau M, Hamraoui A, Bareau I, Blanchard P, Gil-Munoz MI, Barberan FT. Polyphenolic compounds of Mediterranean Lamiaceae and investigation of orientational effects on *Acanthoscelides obtectus* (Say). J Stored Prod Res. 2004; 40(4): 395–408.

[44] AlJabr AM, Hussain A, Rizwan-ul-Haq M, Al-Ayedh H. Toxicity of plant secondary metabolites modulating detoxification genes expression for natural red palm weevil pesticide development. Molecules. 2017; 22(1): 169. 10.3390/molecules22010169 PMC6155707 28117698

