# Correction: Bioactivity-guided isolation of rosmarinic acid as the principle bioactive compound from the butanol extract of *Isodon rugosus* against the pea aphid, *Acyrthosiphon pisum*

**DOI:** 10.1371/journal.pone.0224416

**Published:** 2019-10-22

**Authors:** Saira Khan, Clauvis Nji Tizi Taning, Elias Bonneure, Sven Mangelinckx, Guy Smagghe, Raza Ahmad, Nighat Fatima, Muhammad Asif, Mohammad Maroof Shah

In the title of this article, the word “principle” should be “principal.” The correct title is: Bioactivity-guided isolation of rosmarinic acid as the principal bioactive compound from the butanol extract of *Isodon rugosus* against the pea aphid, *Acyrthosiphon pisum*. The correct citation is: Khan S, Taning CNT, Bonneure E, Mangelinckx S, Smagghe G, Ahmad R, et al. (2019) Bioactivity-guided isolation of rosmarinic acid as the principal bioactive compound from the butanol extract of *Isodon rugosus* against the pea aphid, *Acyrthosiphon pisum*. PLoS ONE 14(6): e0215048. https://doi.org/10.1371/journal.pone.0215048

## References

[1] Khan S, Taning CNT, Bonneure E, Mangelinckx S, Smagghe G, Ahmad R, et al (2019) Bioactivity-guided isolation of rosmarinic acid as the principle bioactive compound from the butanol extract of *Isodon rugosus* against the pea aphid, *Acyrthosiphon pisum*. PLoS ONE 14(6): e0215048 10.1371/journal.pone.0215048 31233534 PMC6590782

