# Supplementary material for: Bioactivity-guided isolation of rosmarinic acid as the principle bioactive compound from the butanol extract of Isodon rugosus against the pea aphid, Acyrthosiphon pisum
Source: PLoS One. 2019 Jun 24;14(6):e0215048. doi: 10.1371/journal.pone.0215048 (PMC6590782; doi:10.1371/journal.pone.0215048)
Supplement: S2 Table — (DOCX) [file pone.0215048.s002.docx]

**S2 Table. Subfractions (1A-14A) collected from the first reversed-phase flash chromatography of butanol extract (500 mg)**

| **Fractions** | **Weight (mg)** |
| --- | --- |
| 1A | 52 |
| 2A | 11 |
| 3A | 46 |
| 4A | 7 |
| 5A | 20 |
| 6A | 15 |
| 7A | 54 |
| 8A | 58 |
| 9A | 14 |
| 10A | 49 |
| 11A | 18 |
| 12A | 15 |
| 13A | 21 |
| 14A | 18 |
