# Supplementary material for: Bioactivity-guided isolation of rosmarinic acid as the principle bioactive compound from the butanol extract of Isodon rugosus against the pea aphid, Acyrthosiphon pisum
Source: PLoS One. 2019 Jun 24;14(6):e0215048. doi: 10.1371/journal.pone.0215048 (PMC6590782; doi:10.1371/journal.pone.0215048)
Supplement: S3 Table — (DOCX) [file pone.0215048.s003.docx]

**S3 Table. Second reversed-phase flash chromatography conditions of butanol fraction (5 g) of *Isodon rugosus***

| **Run Conditions** | |
| --- | --- |
| **Cartridge** | Reveleris 120 g C18 40 µm |
| **Solvent A** | Water |
| **Solvent B** | Methanol |
| **Flow rate** | 85 mL/min |
| **Injection type** | Dry sample |
| **ELSD Carrier** | Isopropanol |
| **Per vial volume** | 25 mL |
| **UV1 Wavelength** | 220 nm |
| **UV2 Wavelength** | 254 nm |
