# Supplementary material for: Bioactivity-guided isolation of rosmarinic acid as the principle bioactive compound from the butanol extract of Isodon rugosus against the pea aphid, Acyrthosiphon pisum
Source: PLoS One. 2019 Jun 24;14(6):e0215048. doi: 10.1371/journal.pone.0215048 (PMC6590782; doi:10.1371/journal.pone.0215048)
Supplement: S4 Table — (DOCX) [file pone.0215048.s004.docx]

**S4 Table. Subfractions (1B-6B) from the second reversed-phase flash chromatography of butanol extract (5 g)**

| **Fractions** | **Weight (mg)** |
| --- | --- |
| 1B | 530 |
| 2B | 830 |
| 3B | 1523 |
| 4B | 195 |
| 5B | 140 |
| 6B | 128 |
